# A pseudogene-signature in glioma predicts survival

**DOI:** 10.1186/s13046-015-0137-6

**Published:** 2015-03-04

**Authors:** Kai-Ming Gao, Xin-cheng Chen, Jun-xia Zhang, Yingyi Wang, Wei Yan, Yong-Ping You

**Affiliations:** Department of Neurosurgery, The First Affiliated Hospital of Nanjing Medical University, Nanjing, China

**Keywords:** Pseudogene, Glioma, Survival, Biomarker

## Abstract

Pseudogene was recognized as a potential tumor suppressor or oncogene in varies of diseases, however its roles in glioma have not been investigated. Our study was to identify the pseudogene-signature that predicted glioma survival. Using a pseudogene-mining approach, we performed pseudogene expression profiling in 183 glioma samples from the Chinese Glioma Genome Atlas (CGGA) and set it as the training set. We found a six-pseudogene signature correlated with patients’ clinical outcome via bioinformatics analyses (P ≤ 0.01), and validated it in the Repository of Molecular Brain Neoplasia Data (REMBRANDT) containing 350 cases. A formula calculating the risk score based on the six-pseudogene signature was introduced and the patients of CGGA set were classified into high-risk group and low-risk group with remarkably different survival (P < 0.001) based on their scores. The prognostic value of the signature was confirmed in the REMBRANDT set. Though the function of these pseudogenes is not clear, the identification of the prognostic pseudogenes indicated the potential roles of pseudogenes in glioma pathogenesis and they may have clinical implications in treating glioma.

## Introduction

Gliomas are the most frequent primary tumors of the CNS (central nervous system) [1], half of which are represented by glioblastoma multiforme (GBM, WHO grade IV), associated with a poor prognosis (median survival less than one year). Identification of markers predicting the survials of gliomas is required for appropriate follow-up and treatment. Over the past few decades, varies of molecular markers were introduced in predicting survival including microRNAs [2], lncRNAs [3] and mutations of unique genes [4]. Accordingly, oligodendrogliomas that show frequent 1p19q co-deletions and mutations of the IDH1 gene are associated with a longer survival than astrocytomas [5]. However, the prognostic significance of pseudogenes in glioma has not been investigated.

In the present study, we identified a prognostic six-pseudogene signature from the CGGA patients set, and validated it in the RAMBRANDT set.

## Materials and methods

### Data set

**Figure 1:**
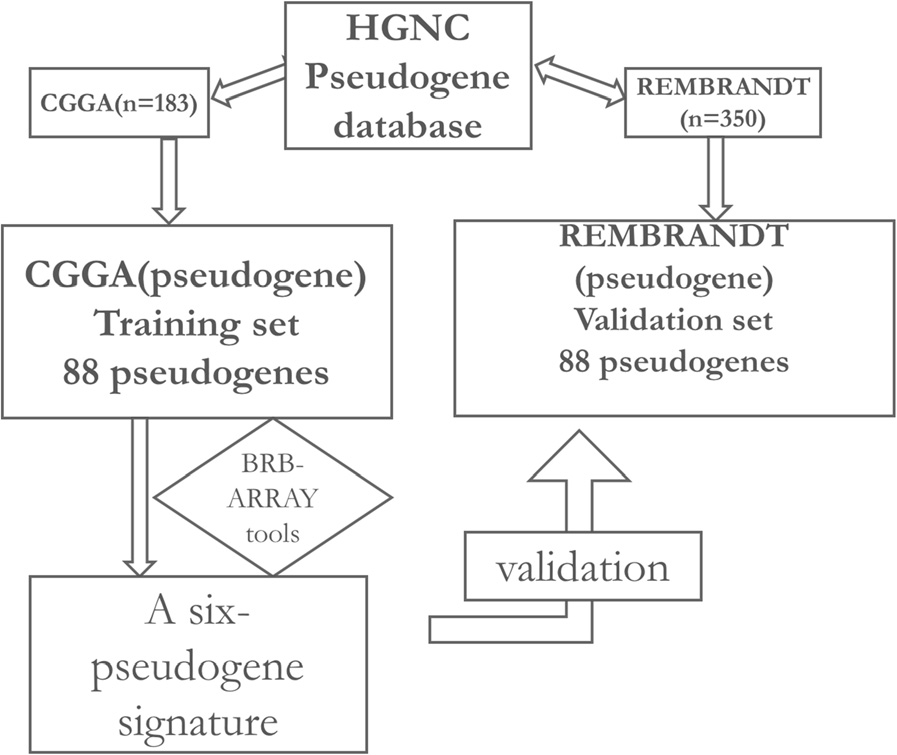
**Flow chart of our study.** Abbreviations: CGGA, the Chinese glioma genome atlas; REMBRANDT, Repository of Molecular Brain Neoplasia Data; HGNC, HUGO Gene Nomenclature Committee; BRB-ARRAY tools, biometric research branch–array tools.

Glioma data sets and corresponding clinical data were downloaded from the publicly available databases, including 183 cases from the Chinese Glioma Genome Atlas (CGGA, http://www.cgcg.org.cn/) and 350 cases from the Repository of Molecular Brain Neoplasia Data (REMBRANDT; http://caintegrator.nci.nih.gov/rembrandt/). Besides, 21 cases without tumors and 102 cases with insufficient data from the REMBRANDT were excluded. Psuedogene gene database was downloaded from the HGNC (HUGO Gene Nomenclature Committee, www.genenames.org), including 12440 pseudogenes, which were cross-matched with the CGGA and the REMBRANDT data set using Microsoft Excel (Microsoft Inc. Redmond, USA). Figure 1 depicts the flow chart of the study.

### Statistical analysis

Pseudogenes represented in both the CGGA set and the REMBRANDT set were identified. Gene expression values represented by multiple probes were collapsed by taking the mean value of the probes [6,7]. The association between the pseudogene expression and patient’s overall survival was assessed by univariable Cox regression analysis along with a permutation test using Biometric Research Branch-Array (BRB-Array) Tools [8]. Genes were considered statistically significant if their permutation P values were less than or equal to 0.01. A risk score formula was established by including each selected pseudogene weighting by its coefficient obtained from multivariable Cox regression analysis [9,10]. Kaplan-Meier survival analysis was used to estimate the survival distributions. The log-rank test was used to assess the statistical significance between stratified survival groups using the median value as the cutoff. A 2-sided P value <0.05 was regarded as significant.

## Results

### Identification of prognostic pseudogenes from the CGGA data set

A total of 88 pseudogenes were identified, which were included in both the CGGA and REMBRANDT gene set. The 183 CGGA patients was set as the training set, which was used for the detection of the prognostic pseudogenes. By using the BRB-Array tools, the univariable Cox proportional hazards regression analysis was done on the pseudogenes expression data, and a six-pseudogene signature relating to overall survival was identified (p ≤ 0.01). Of these, a hazard ratio above 1 indicated that a high level of expression of a gene was associated with shorter survival (SP3P, ANXA2P3, PTTG3P, LPAL2, CLCA3P). A hazard ratio below 1 indicated that a high level of expression of a gene was associated with longer survival (TDH). The results were shown in Table 1.

**Table 1:** **Six pseudogene symbols**

**Gene symbol** ^**c**^	**Parent gene** ^**c**^	**Permutation p-value** ^**a, b**^	**Hazard ratio**	**Coefficient**
SP3P	Sp3	0.001	1.785	0.097
PTTG3P	PTTG1	0.0056	1.365	0.722
LPAL2	LPA lipoprotein	0.0033	1.48	0.148
CLCA3P	CLCA3	0.0044	1.359	−0.185
TDH	TDH	0.0047	0.733	−1.052
ANXA2P3	ANXA2	0.0111	1.31	0.656

^a^Derived from the univariable Cox proportional hazards regression analysis in the 183 training-set patients.

^b^Obtained from permutation test repeated 10,000 times.

^c^Detailed function report was described in the Discussion section.

### The six-pseudogene signature correlates with patients’ survival

**Figure 2:**
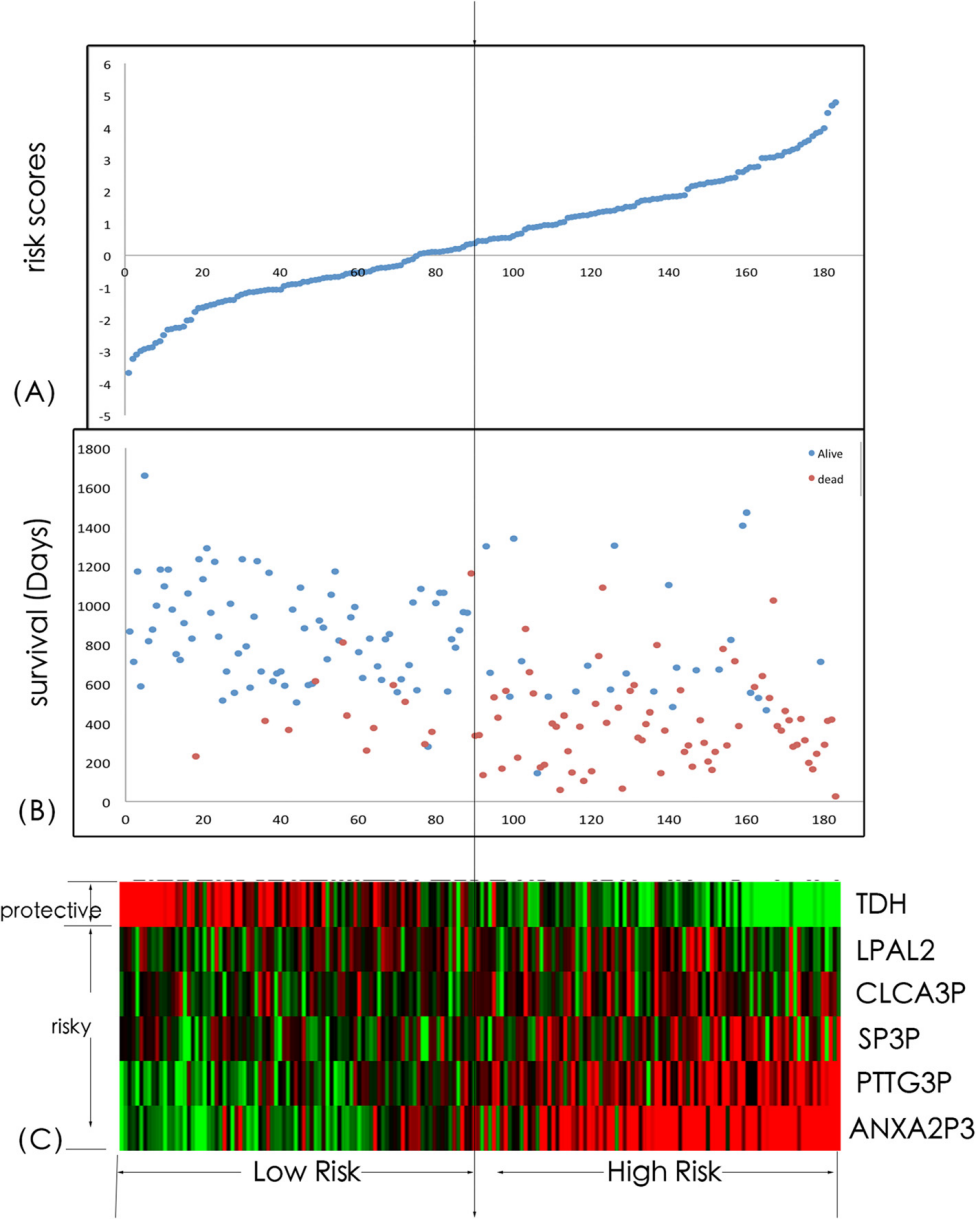
**Pseudogene risk score analysis of CGGA patients.** The distribution of six-pseudogene risk score, patients’ survival status and pseudogene expression signature were analyzed in the entire CGGA patients (n = 183). The vertical axis in **(A)** means risk score. The vertical axis in **(B)** means survival time (days). The dotted line in the middle divided the patients into two groups – one group with low risk score and the other one with high score. As the risk score rising, the patients had a shorter survival time. As the risk score rising, the expression value of TDH lowered, and the other 5 pseudogenes’ ascended, which meant the TDH was a protective one, and the other 5 were risky. **(A)** Pseudogenes risk score distribution; **(B)** Patients’ survival status and time; **(C)** Heatmap of the pseudogene expression profiles. Rows represent pseudogenes, and columns represent patients. The black dotted line represents the median pseudogenes risk score cutoff dividing patients into low-risk and high-risk groups.

A risk-score formula was established according to the expressions of these selected pseudogenes and their coefficients, as follows: Risk Score = (0.097*expressing values of SP3P + 0.722*expressing values of PTTG3P + 0.149*expressing values of LPAL2 + (-0.185*expressing values of CLCA2P) + (-1.052*expressing values of TDH) + 0.656*expressing values of ANXA2P3). We then calculated the six-pseudogene signature risk score for each patient in the CGGA set, and ranked them due to their scores. The 183 patients were divided into a low-risk group and a high-risk group using the median risk score as the cutoff point. Patients in the low-risk group had remarkable longer overall survival time than those in the high-risk group (P < 0.001). Our result was shown in Figure 2.

### Validation of the six-pseudogene signature in predicting survival in the REMBRANDT dataset

To confirm our findings, we validated our six-pseudogene signature in the REMBRANDT set. After calculating risk score of all the patients using the formula in the REMBRANDT set, the patients were assigned into a low-risk group and a high-risk group using the same cutoff point as for the CGGA set. Patients in the low-risk group had significant longer over survival time than those in the high-risk group (P < 0.001), which also maintained the consistence with the CGGA set.

**Figure 3:**
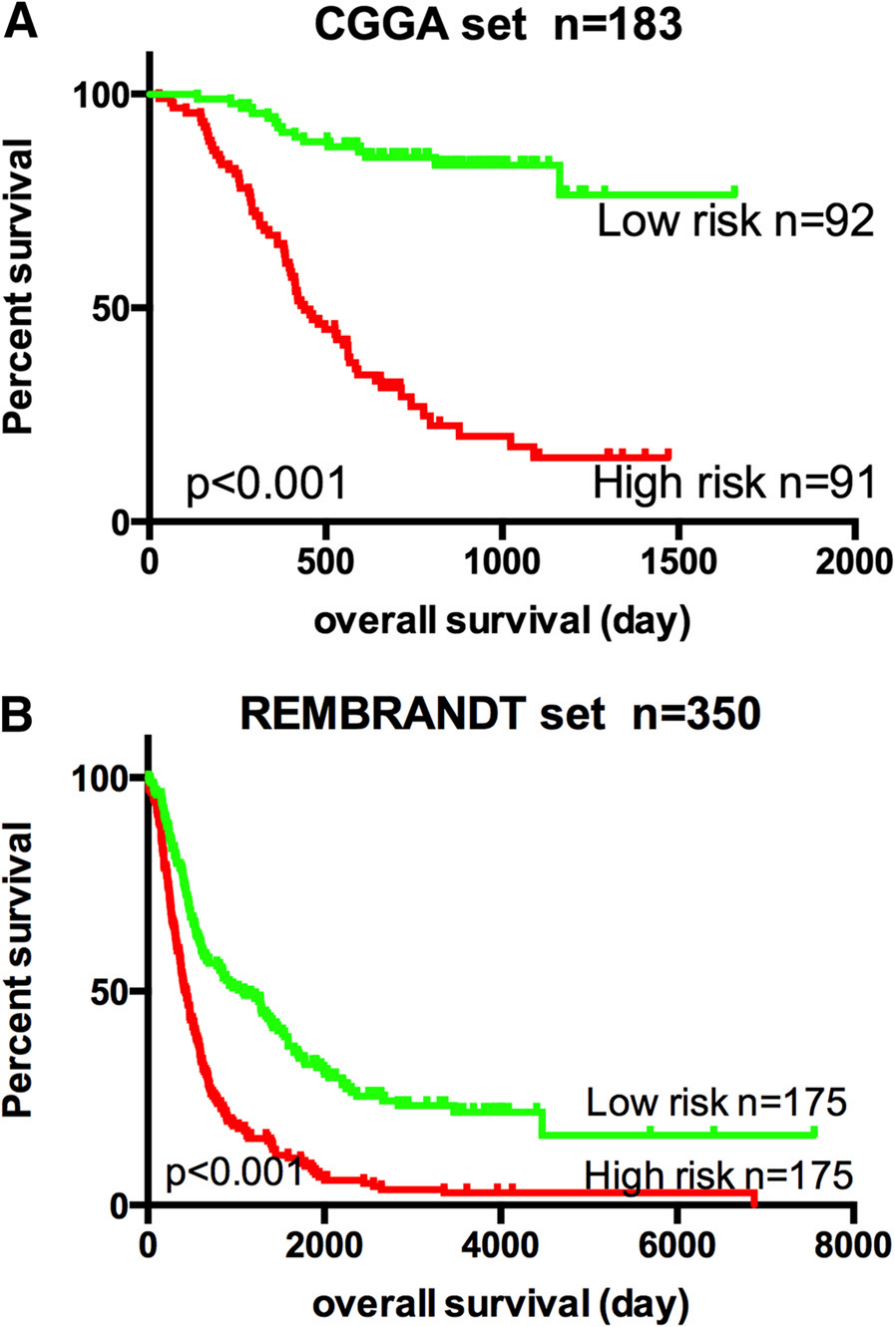
**Kaplan–Meier estimates of the overall survival of CGGA patients using the six-pseudogene signature.** The Kaplan–Meier plots were used to visualize the survival probabilities for the low-risk versus high-risk group of CGGApatients determined on the basis of the median risk score. **(A)** Kaplan–Meier curves for CGGA training-set patients (n = 183); **(B)** Kaplan–Meier curves for REMBRANDT patients (n = 350); The tick marks on the Kaplan–Meier curves represent the censored subjects. The differences between the two curves were determined by the two-sided log-rank test. The number of patients at risk was listed below the survival curves.

In both of the CGGA set and the RAMBRANDT set, we found that the patients of the high-risk group tended to express high levels of risky pseudogenes (SP3P, ANXA2P3, PTTG3P, LPAL2, CLCA3P), whereas the patients of the low-risk group tended to express high level of protective pseudogenes (TDH). Results were shown in Figure 3.

### The six-pseudogene signature was independent of age and gender

**Figure 4:**
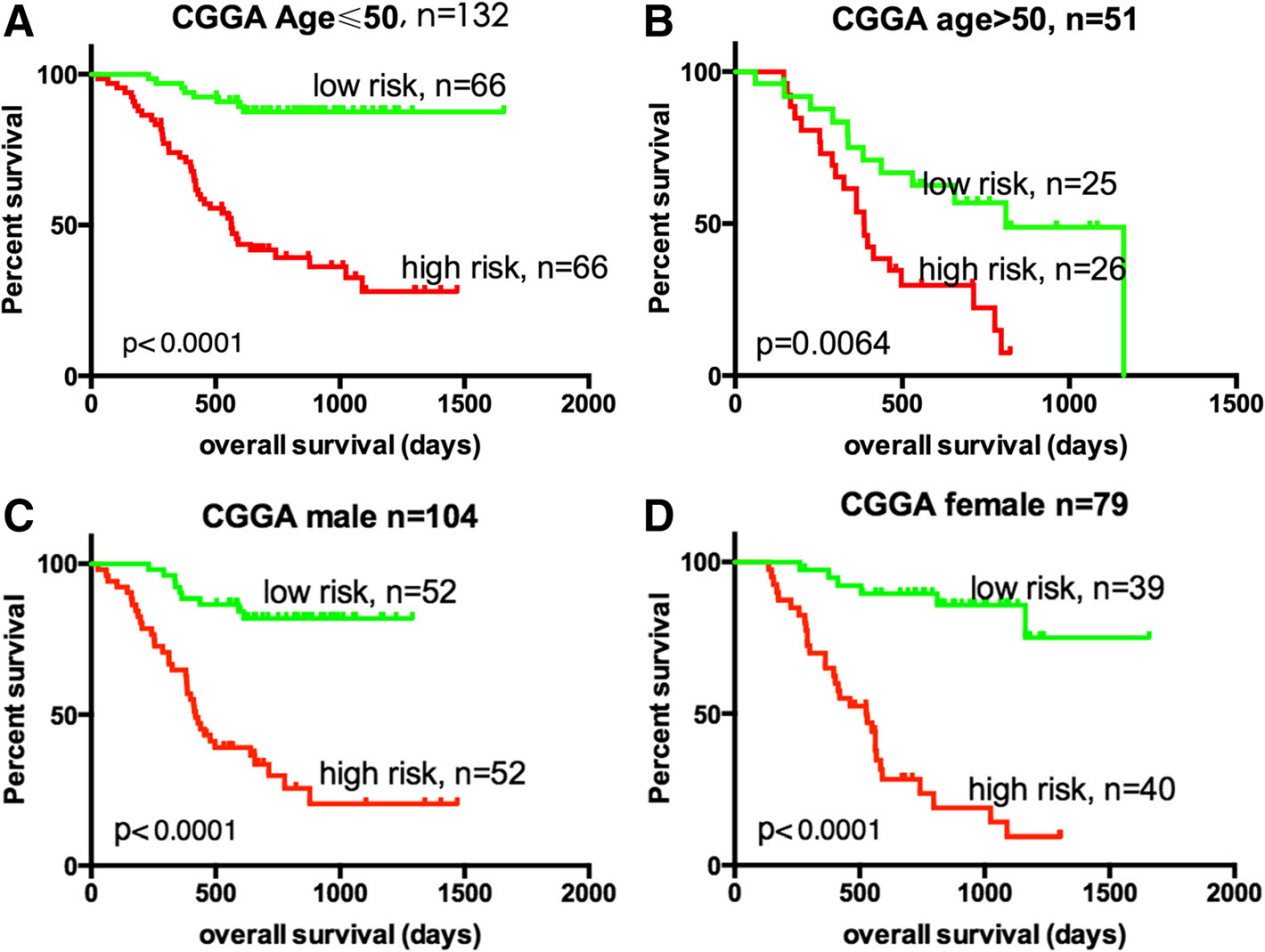
**Kaplan–Meier estimates of the overall survival of CGGA patients using the six-pseudogene signature, stratified by age.** Entire CGGA patients (n =183) were first stratified by age (age ≤ 50 or >50). Kaplan–Meier plots were then used to visualize the survival probabilities for the low-risk versus high-risk group of patients determined on the basis of the median risk score from the training-set patients within each age group. **(A)** Kaplan–Meier curves for younger TCGA patients (age ≤ 50, n = 132); **(B)** Kaplan–Meier curves for elder CGGA patients (age > 50, n = 51). **(C)** Kaplan–Meier curves for CGGA patients (male, n = 104); **(D)** Kaplan–Meier curves for CGGA patients (female, n = 79). The tick marks on the Kaplan–Meier curves represent the censored subjects. The differences between the two curves were determined by the two-sided log-rank test. The number of patients at risk was listed below the survival curves.

We stratified the entire CGGA patients (n = 183) into a younger group (age ≤ 50) or an elder one (age > 50). This analysis showed that within each age group, the six-pseudogene risk score could further subdivide the patients into those likely to have longer survival and those likely to have shorter survival. Then, the entire CGGA group was subdivided into 2 groups based on gender. Both in the male group and female group, the patients with lower risk scores had longer overall survival. These results were shown in Figure 4(A-D).

**Figure 5:**
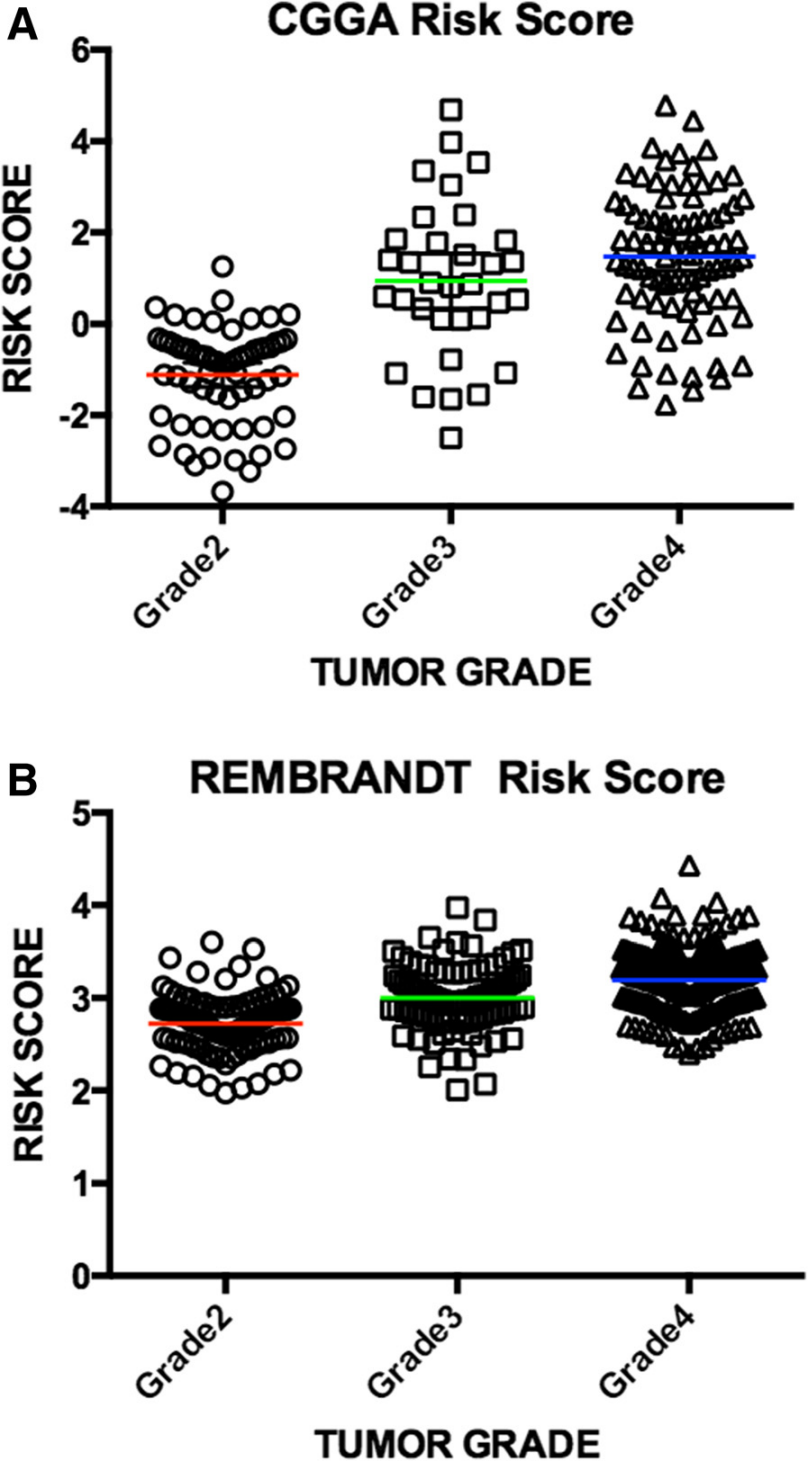
**Risk score correlate with tumor malignance. (A)** In CGGA set, mean risk score rose as the tumor malignance ascending. P value of A-nova test was less than 0.001, which meant the mean risk score differed from tumor grades significantly. **(B)** In REMBRANDT set, mean risk score rose as the tumor malignance ascending. P value of A-nova test was less than 0.001, which meant the mean risk score differed from tumor grades significantly.

Besides, we made an analysis on relationship between the six-pseudogene signature and WHO Grade, which was showed in Figure 5. We can conclude that mean risk score ascended with the tumor malignance in both the CGGA set and the REMBRANDT set.

**Figure 6:**
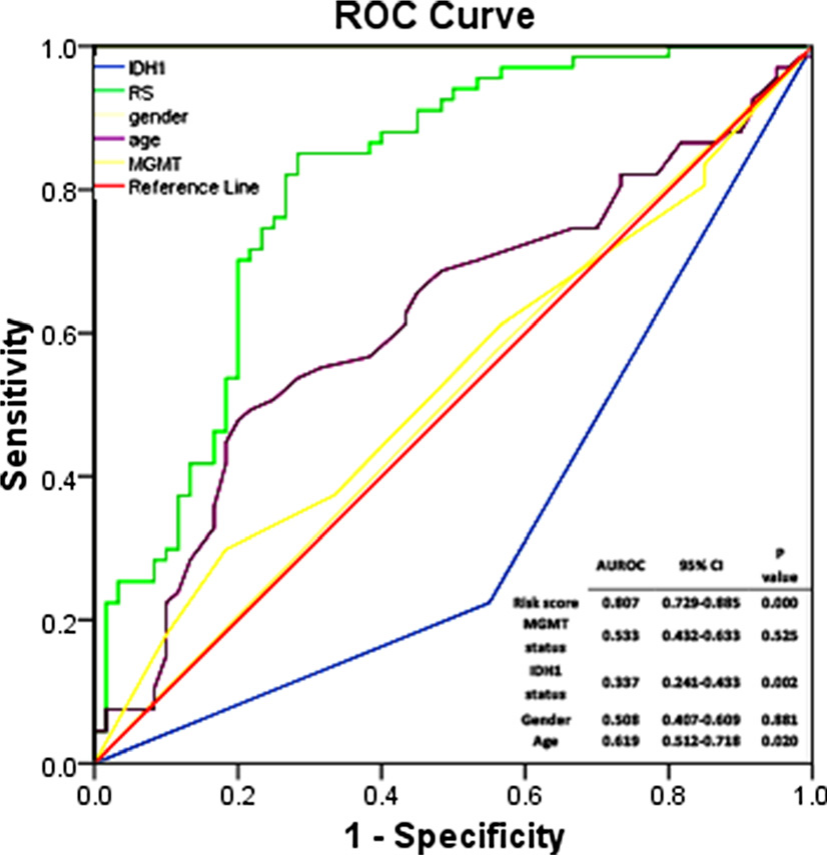
**ROC analysis of the sensitivity and specificity of the overall survival prediction by the six-pseudogene risk score, IDH1 status, MGMT expression level, gender and age in CGGA data set.** P values were from the comparisons of the area under the ROC (AUROC) of six-pseudogene risk score versus those of MGMT expression level, IDH1 status, gender and age, respectively. As can be seen, the six-pseudogene risk score showed a better prediction of overall survival than age, IDH1 status, and MGMT expression level.

We also performed receriver operating characteristic (ROC) analysis to compare the sensitivity and specificity of survival prediction between the six-pseudogene model, MGMT expression status, age, gender, and IDH1 status. The area under receiver operating characteristic (AUROC) was determined and compared between these five prognostic factors. As shown in Figure 6, the AUROC of the six-pseudogene risk score was 0.807, which was significantly larger than that of age (AUROC = 0.619, P = 0.020) and gender (AUROC = 0.508, P = 0.881); when compared with the MGMT status and IDH1 status, the AUROC of the six-pseudogene risk score was larger than theirs’. (For IDH1, 0.807 versus 0.377, P = 0.049; for MGMT, 0.807 versus 0.533, P = 0.051). These results indicated that six-pseudogene signature might have a better predictive ability in predicting worse prognosis (Figure 6).

## Discussion

Pseudogenes are believed to be dysfunctional genes that have lost their protein-coding ability or are otherwise no longer expressed in the cell [1]. However, some of them do play important roles in regulating their parent genes, and some even can be transcribed into RNA. It is also recognized that pseudogenes may regulate tumor suppressors and oncogenes. Pseudogenes were reported to correlate with varies of diseases, such as CYP4Z2P related to breast cancer [11], while it has not been investigated in glioma.

To verify our hypothesis, we performed pseudogene profiling in large cohorts of glioma patients from CGGA and REMBRANDT. By analyzing the association between gene expression profiling and clinical outcome of glioma patients, we identified a six-pseudogene signature significantly related to the overall survival of glioma patients.

### Functional characteristics of the six pseudogenes

As for the functional characteristics of the six pseudogenes, one pseudogene called TDH, or L-threonine dehydrogenase, was believed to be protective, whereas the other five were risky ones.

TDH is a pseudogene of L-threonine 3-dehydrogenase (TDH), whose expression was found in human tissues including heart, brain, placenta, lung, liver, skeletal muscle, kidney, pancreas, spleen, thymus, prostate, testis, ovary and small intestine. TDH mRNA was present in most cell types examined, but was below the level of detection in endothelial cells, glioma cell lines and some leukaemia cell lines according to the study of Alasdair J Edgar [12]. Though TDH is an expressed pseudogene, its function is not clear. So far, we can’t find a single research on TDH. However, in our study, the high level expression of TDH related with longer overall survival, which indicated TDH might be a protect pseudogene. As the expression of TDH mRNA was low in glioma cell line [12], we conjectured that TDH might serve as an endo-generate siRNA, or work as a molecular sponge combining microRNAs. The mechanism is subject to be investigated.

The present study demonstrated the associations between the high expressions of these five pseudogenes and shortened overall survival. The roles of these pseudogenes in glioma pathogenesis are presently unclear, and our findings suggest that they deserve further studies. We might conjecture some information from their parent genes that were studied before. The parent genes of these pseudogenes (SP3P, ANXA2P3, PTTG3P, LPAL2, CLCA3P) are SP3 transcription factor (Sp3), Annexin A2 (ANXA2), pituitary tumor-transforming 1(PTTG1), Lp(a)(LPA lipoprotein) and chloride channel accessory 3 (CLCA3) respectively.

ANXA2 was found expressed significant higher in NSCLC (Non-small cell lung cancer) tissue compared to that in adjacent non-cancerous tissue according to the study of Jia et al. [13], and Zhang et al. found annexin A2 silencing inhibits invasion, migration, and tumorigenic potential of hepatoma cells [14], which indicated that annexin A2 (ANXA2) might serve as an important mediator of malignant transformation and development of hepatocellular carcinoma. The pseudogene of ANXA2 may paly the same role in glioma.

PTTG is an oncogene that plays diverse roles in the occurrence, proliferation, and invasion of a variety of tumors, and PTTG has already served as one of the markers of proliferative activity progress in many tumors [15]. Study were carried out on the association between PTTG expression level and human pituitary macroadenomas [16], which suggested PTTG may promote invasive tumor growth by stimulating pituitary adenomas proliferation. As the pseudogene of PTTG, PTTG3P may play as an oncogene in glioma, which played as a risky gene.

CLCA may serve as a tumor suppressor, which cound be inferred from the result that hCLCA2 is expressed in normal breast epithelium but not in 29 breast cancer lesions of different stages [17,18]. Our research showed CLCA3P was a risky pseudogene. We conjectured that CLCA3P might be a molecular sponge same as PTENP1. However, the mechanism is subject to be investigated.

The encoded protein of Lp(a) constitutes a substantial portion of lipoprotein(a) and is proteolytically cleaved, resulting in fragments that attach to atherosclerotic lesions and promote thrombogenesis [19,20] which seemed to have no relationship with tumor.

### Limitations of the study

Our study does have some small limitations. In our study, the data sets were not designed for detecting pseudogenes, so, only a small minority of human pseudogenes (88 genes) were included in the analysis. Because of the different platform using in the CGGA and REMBRANDT, we only retained the pseudogenes that appeared on both platforms for survival analysis. So, these prognostic pseudogenes identified here might not represent all the pseudogenes correlated with glioma overall survival. Besides, the mechanisms of the pseudogenes are subject to be investigated on which we intended to study in our later research. However, the significant and consistent correlation of our six-pseudogene signature with overall survival in several independent data sets indicates that it is a potentially powerful prognostic marker for glioma.

In conclusion, by employing two independent patients cohorts, our study revealed the prognostic values of pseudogenes in glioma for the first time. Our findings strongly prompt that pseudogene signatures may be of use in predicting the treating outcome and may be novel biomarkers in glioma prognoses. As the updating of common databases, pseudogenes will show more value in future glioma studies, while this is what we continue to explore in the future. We will focus on the function of the pseudogenes we found and try to validate our findings in our clinical outcome.
